# Design the magnetic microencapsulated phase change materials with poly(MMA-MAA) @ n-octadecane modified by Fe_3_O_4_

**DOI:** 10.1038/s41598-018-34583-5

**Published:** 2018-11-06

**Authors:** Xueheng Zhuang, Ying Zhang, Chang Cai, Jing Zhang, Yuejin Zhu

**Affiliations:** Department of Microelectronic Science and Engineering, Ningbo Collaborative Innovation Center of Nonlinear Calamity System of Ocean and Atmosphere, Ningbo University, Zhejiang, 315211 China

## Abstract

Magnetic microencapsulated phase change materials (magnetic MicroPCMs) are hotly researched for their dual-functions with phase change and magnetic properties, which provided the new applications in fields of maneuverable phase change materials and infrared electromagnetic dual shield. A series of magnetic MicroPCMs samples are synthesized by polymerization and coprecipitation method and the chemical composition contained poly(MMA-MAA) @ n-octadecane modified by Fe_3_O_4_. In addition, the characterizations exhibit the excellent magnetic and phase change properties. The magnetic MicroPCMs samples present 20 emu·g^−1^ saturation magnetization with still high enthalpy of 132 J·g^−1^, which fully illustrates that the magnetic MicroPCMs fulfill both application on thermal energy storage and magnetic control.

## Introduction

Phase Change Materials (PCMs) have been attracting increasing attention owing to their excellent thermal properties. Take n-octadecane for instance, a single kilogram of n-octadecane can absorb or release energy about 228 kJ as its phase change occurs isothermally^1^, which can improve the temperature of one-kilogram water from 1 °C to 55 °C. PCMs, hence, has been widely used in many fields, such as solar energy storage^2–4^, construction energy conservation^5–8^, thermal regulated clothes^9,10^, Electro-to-Heat Conversion^11–13^. In general, There are three categories of PCMs, organics, salt hydrates and eutectic compounds^14^. N-octadecane as a kind of organic PCMs has been focused more owing to its high enthalpy and near room phase change temperature (28 °C).

To overcome the mobility of liquidus PCMs, encapsulating phase change materials become the hot topic in research of PCMs. A variety of packaging process have been applied on the encapsulated phase change materials, such as PCMs composite fibers^9,10,15^, tube encapsulation^16,17^, PCMs in panel^18,19^, PCMs in pouches^20^, PCMs/Graphite compound^12,21–23^, and PCMs microencapsulation^24,25^. Among these packaging technologies, microencapsulation as an important packaging technology toward PCMs has been well developed. Both of organic and inorganic materials can be used as shell materials. Organic materials such as polymer are commonly used in those materials and they are easily prepared by polymerization such as polystyrene^26^, polyurea-formaldehyde resin^27^, melamine-formaldehyde resin^28^, and poly(methyl methacrylate) (PMMA)^25,29^. As one of the polymers, poly(MMA-MAA) presents a stable reaction process and a higher microencapsulated efficiency with formaldehyde free^25^.

Nowadays, more and more functions of microencapsulated phase change materials have been reported, such as reversible thermochromic property^30^, antibacterial and thermoregulation^31^, and photo-thermal conversion performance^32^. Meanwhile, electronic device has already found the widespread application in various fields. But enormous heat and electromagnetic radiation are produced during the electronic devices usage and significantly impact the performance and the safety of user. Magnetic MicroPCMs can avoid producing enormous heat and electromagnetic radiation because of its latent heat and electromagnetic shielding. Furthermore, the magnetic MicroPCMs can be controlled by magnet or electromagnetic device when used to MicroPCMs thermal fluid. There are some publications concerning the magnetic MicroPCMs just with silica or organosilicon materials shell^33–35^. However, poly(MMA-MAA) based magnetic MicroPCMs have been reported rarely because Fe_3_O_4_ could not be well contained in n-octadecane owing to the poor dispersion of Fe_3_O_4_ in oil. Here, we change the idea and encapsulated n-octadecane to poly(MMA-MAA) firstly, then Fe_3_O_4_ was coprecipitated on the surface of MicroPCMs after SDS modified. At last, the magnetic MicroPCMs was obtained and characterized.

## Methods

### Materials

n-octadecane (PCMs, purity 99 wt.%), Sodium dodecyl sulfate (SDS, as dispersant. AR, 92.5–100.5 wt.%), Methyl methacrylate (MMA was used as monomer of the polymerization, purity 99.5 wt.%), Methylacrylic acid (MAA, as monomer of the polymerization, purity 98 wt.%), Benzoyl peroxide (BPO was used as an initiator, AR), Potassium persulfate (K_2_S_2_O_8_ was used as a secondary initiator, ACS, purity 99.0 wt.%(RT)), Iron chloride hexahydrate (FeCl_3_·6H_2_O, purity 99 wt.%), Iron chloride tetrahydrate (FeCl_2_·4H_2_O, purity 99.95 wt.%) and ammonia solution (NH_3_·H_2_O, concentration 25–28 wt.%) were commercially obtained from Aladdin Industrial Corporation, Shanghai, China.

### Synthesis

#### Synthesis of MicroPCMs

A 250 mL three-neck round-bottomed flask was equipped with a mechanical stirrer, a reflux condenser and a nitrogen gas inlet tube, which was put in a water thermostat bath as a reaction vessel. 0.25 g SDS as a dispersant dissolve in 100 mL distilled water, constitute the water phase. Oil phase consist of 9 g n-octadecane, 4 g MMA, 2 g MAA, and 0.1 g BPO. The water phase was added to the 250 mL three-neck round-bottomed flask is ready, and then was heated to 55 °C with stirring to 500 rpm for 10 min. Afterwards, the oil phase was poured into the water phase to form an oiliness suspension with stirring to 1200 rpm for 15 min. And then, the suspension was heated to 85 °C for 3.5 h with rotational rate was turned down 800 rpm, the nitrogen gas inlet tube was opened at the same time. 0.1 g K_2_S_2_O_8_ was dissolved into 1.4 g of water to form an aqueous solution which was added into the reaction suspension and continued for 1.5 h with the same stirring rate after all. Ultimately, the final suspension was separated with vacuum filtration after washed by distilled water at 50 °C to eliminate impurities and a wet cake was dried in an oven at 50 °C for 48 h to remove the residual water. Finally, white particles were obtained which like powder named MicroPCMs^25^.

#### Synthesis of magnetic MicroPCMs

A 250 mL three-neck round-bottomed flask was equipped equally. 2 g of the resultant MicroPCMs was added into the three-neck round-bottomed flask with 50 mL distilled water, 0.05 g SDS was added as the surfactant. After the mixture was heated to 40 °C with stirring to 300 rpm for 15 min to obtain the homogeneous dispersions. Then, 0.88 g FeCl_2_·4H_2_O and 0.6 g FeCl_3_·6H_2_O was added into the dispersions with nitrogen gas inlet tube was opened until them fully dissolved. Afterward, about 20 mL NH_3_·H_2_O was dropped into the dispersions at *pH* 10.0–11.0. Black microparticles were obtained after 10 min. Finally, the black microparticles were carefully collected by a magnet and were washed with distilled water several times. The obtained microparticles were filtrated and dried in an oven at 50 °C for 24 h. The final products were prepared for further characterization and testing.

### Characterization

The surface morphology and elemental compositions of the magnetic MicroPCMs were presented using a Hitachi S-4800 scanning electron microscope (SEM) equipped with an energy-dispersive X-ray (EDX) spectroscopy. Tecnai F20 was chosen to characterize diameter and microstructure of fabricated Fe_3_O_4_ as the HRTEM. The chemical structures of the samples were investigated using a Nicolet 6700 FTIR spectrophotometer, and the simples were measured at a wave number of 4000–400 cm^−1^ using a KBr sampling sheet. A Germany Bruker D8 Advance X-ray diffractometer with Cu-Ka radiation (*λ* = 0.154 nm) gave the Powder X-ray diffraction (XRD) data at a scan rate of 10°/min in the 2*θ* range of 5°–90°. The phase-change behaviors of the samples were investigated using a PerkinElmer Diamond Dynamic differential scanning calorimetry (DSC) scans, coupled with a thermal analysis data station. All of the measurements were carried out under a nitrogen atmosphere at a heating or cooling rate of 10 °C/min in the range of 0–60 °C, and the mass for each sample was about 7–8 mg. Thermogravimetric analysis (TGA) of the magnetic microcapsules was carried out using a TA Instruments SDT Q600 thermal gravimetric analyzer under a nitrogen atmosphere. The sample with a mass of about 7–8 mg was placed in an aluminum crucible and then was ramped from room temperature up to about 600 °C at a heating rate of 10 °C/min. A Physical Property Measurement System (PPMS-9, Quantum Design) performed the magnetic properties of the samples in applied fields up to 20,000 Oe at room temperature (~298 K). The distribution of the particle diameter of magnetic MicroPCMs was measured on the Microtrac S3500 SI particle size analyzer, with the specimens were dispersed in ethanol.

## Results and Discussions

### Synthetic strategy

**Figure 1:**
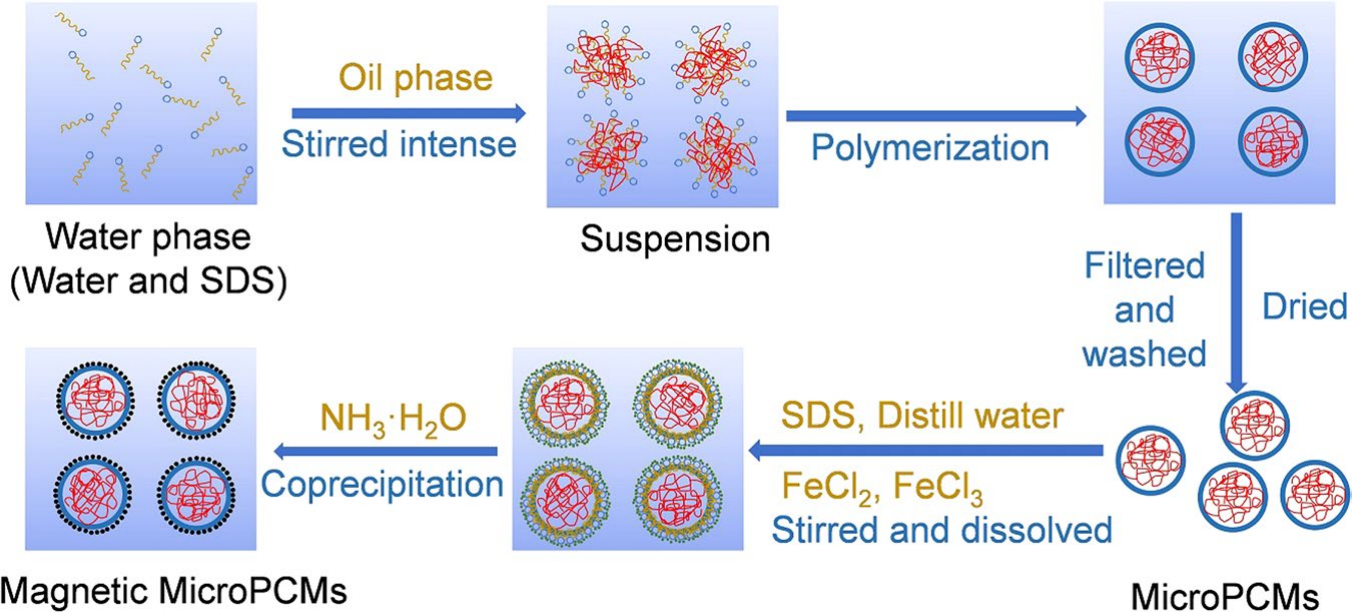
Schematic synthetic strategy of the magnetic MicroPCMs with an n-octadecane core and Fe_3_O_4_/poly(MMA-MAA) shell.

The synthetic method of the magnetic MicroPCMs with Fe_3_O_4_ is shown in Fig. 1. The MicroPCMs was fabricated by n-octadecane as the core and poly(MMA-MAA) as the shell firstly in this work. After the MicroPCMs was manufactured, a little of SDS was chosen to modify the surface of MicroPCMs. Then the Fe^2+^ and Fe^3+^ were dissolved and attached to MicroPCMs due to the amphipathic character of SDS, ammonia was dropped in reaction systems as precipitating reagent. And then, the Fe_3_O_4_ particles were produced adopted coprecipitation method and attached to the shell of the MicroPCMs owing to physical adsorption. The SDS was used as surface modifers to prevent the Fe_3_O_4_ particles getting off the surface of MicroPCMs. The chemical equation for fabrication of Fe_3_O_4_ is shown as below:

$${{\rm{Fe}}}^{2+}+{{\rm{2Fe}}}^{3+}+{{\rm{8NH}}}_{{\rm{3}}}\cdot {{\rm{H}}}_{{\rm{2}}}{\rm{O}}={{\rm{Fe}}}_{{\rm{3}}}{{\rm{O}}}_{{\rm{4}}}\downarrow +{{{\rm{8NH}}}_{4}}^{+}+{{\rm{4H}}}_{{\rm{2}}}{\rm{O}}$$

Molar ratio of Fe^2+^ and Fe^3+^ is 1:2 in theory according to the equation. But the ratio is 2:1 or 3:1 in actual experiments because the Fe^2+^ was easy to be oxidized^36^.

### Optimum Condition of MicroPCMs’ Fabrication

The dosage of copolymer poly(MMA-MAA) was determined refer to the reference^25^. The mass ratio of n-octadecane and poly(MMA-MAA) was 9:6, as change of the dosage of the dispersant SDS, there are different heat characters of MicroPCMs. Therefrom the actual average enthalpy (*ΔH*_*a*_) of the phase change process will be compared clearly.

*ΔH*_*a*_ = (*ΔH*_*c*_ + *ΔH*_*m*_)/2, *ΔH*_*c*_ means the crystallization enthalpy of the n-octadecane, *ΔH*_*m*_ means the melting enthalpy of the n-octadecane.

**Figure 2:**
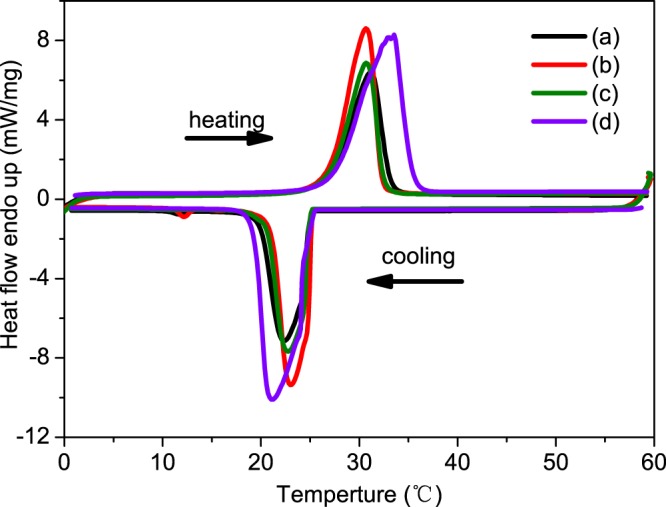
DSC cooling and heating thermograms of (**a**) SDS 0.5g, (**b**) SDS 0.25g, (**c**) SDS 0.125g, (**d**) pure n-octadecane.

The measured enthalpy, melting peak temperatures and crystallization peak temperatures of the MicroPCMs and pure n-octadecane are listed in Table 1 and the DSC curves are shown in Fig. 2. Compared the average enthalpy to pure enthalpy, the MicroPCMs are lower universally due to the content decrease of the n-octadecane (as Table 1 indicated). Moreover, the melting peak temperature was shifted left and the crystalline temperature was shifted right compare with pure n-octadecane, which means the thermal conductivity of the MicroPCMs is higher than pure n-octadecane’s. It is observed that pure n-octadecane exhibits single peak at 21.1 °C in its solidification process. However, there are two peaks at 22.5 °C and 11.7 °C in the crystallization process of the MicroPCMs due to the impurity induced heterogeneous nucleation in the solidification process^37^.

**Table 1:** Thermal properties of MicroPCMs with various SDS dosage.

SDS (g)	*T*_*m*_ (°C)	Δ*H*_*m*_ (J·g^−1^)	*T*_*c*_ (°C)	Δ*H*_*c*_ (J·g^−1^)	Δ*H*_*a*_ (J·g^−1^)	n-octadecane (wt.%)
0.5	30.3	146.7	23.5	139.4	143.0	60.1
0.25	30.3	176.4	23.5	165.6	171	71.9
0.125	30.3	145.9	23.5	141.9	143.9	60.5
0	33.3	237.2	21.1	238.6	237.9	100

**Figure 3:**
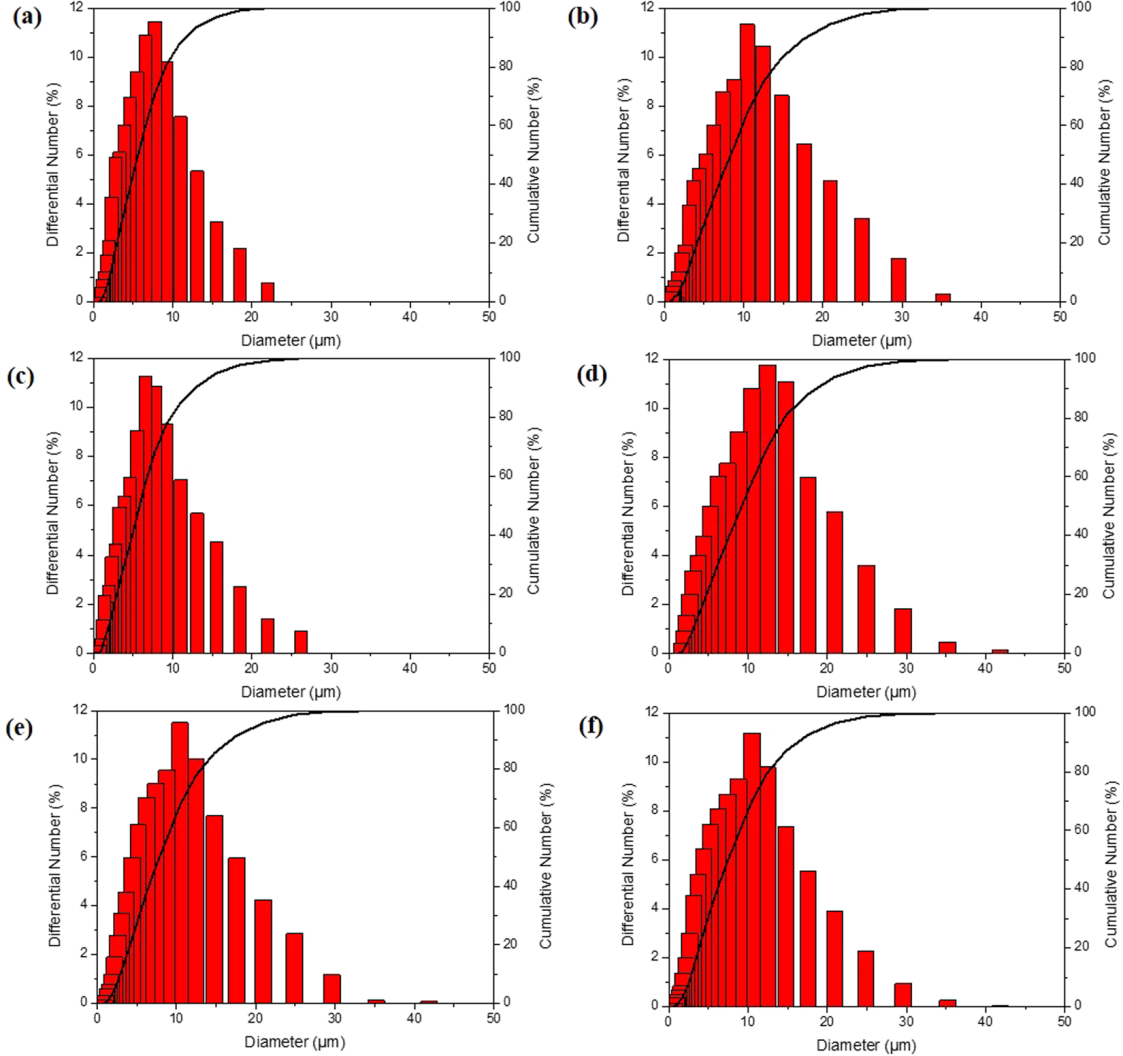
The size distributions of the MicroPCMs of (**a**) SDS 0.5g, (**b**) SDS 0.25g, and (**c**) SDS 0.125g; and magnetic MicroPCMs with Fe_3_O_4_/MicroPCMs mass ratios (**d**) 70/30, (**e**) 20/80, and (**f**) 11/89.

It’s also clearly shown that there are dramatic effects of different dosage of SDS on the content n-octadecane in Fig. 2. The *ΔH*_*a*_ of the MicroPCMs were 143.0, 171 and 143.9 J·g^−1^ when synthesized with the SDS mass of 0.5, 0.25 and 0.125 g, respectively. The enthalpy was increased firstly and then decreased with the decreased in the mass of SDS. The distributions of the particle diameter of MicroPCMs fabricated with different masses of SDS were shown in Fig. 3(a–c). When the mass of SDS is 0.5 g, the diameters of the specimen are in the range of 0.8–22 μm, and the average diameter is about 8 μm. When the mass of SDS is decreased to 0.25 g, the diameters of the specimen are in the range of 0.9–35 μm, and the average diameter is about 11 μm. When the mass of SDS is decreased to 0.125 g, the diameters of the specimen are in the range of 0.8–26 μm, and the average is about 7 μm. The diameter ranges of the MicroPCMs with 0.5 g SDS additive as dispersant is the narrowest, but the average diameter reached the maximum when the mass of dispersant SDS is 0.25 g. The dosage of dispersant SDS influences on the sizes of droplet in the suspension, and then affects the diameter of the microcapsules after the polymerization. It has been reported that the amount of the dispersant would make a great influence on the particle diameter of the MicroPCMs. Because the thickness of shell is tiny, the size distribution of particles can be changed largely by the shell’s structure^38^. The PMMA-MAA structure could be changed by the amounts of SDS, as different amounts of surfactant were added in the same amounts of reactive monomers, the structure of copolymers will be changed in polymerization. In addition, the amounts of SDS would influence on the size of floating droplet in the suspension polymerization. And the particle diameter curve appeared inverted U-shaped with the increasing of the amount of the dispersant. As the increasing of the particle diameter, the content of the n-octadecane will increase and lead to raise of the enthalpy^38,39^. In this paper, the magnetic MicroPCMs was fabricated from the MicroPCMs with the maximum enthalpy.

### The morphology, crystallinity and composition of magnetic MicroPCMs

**Figure 4:**
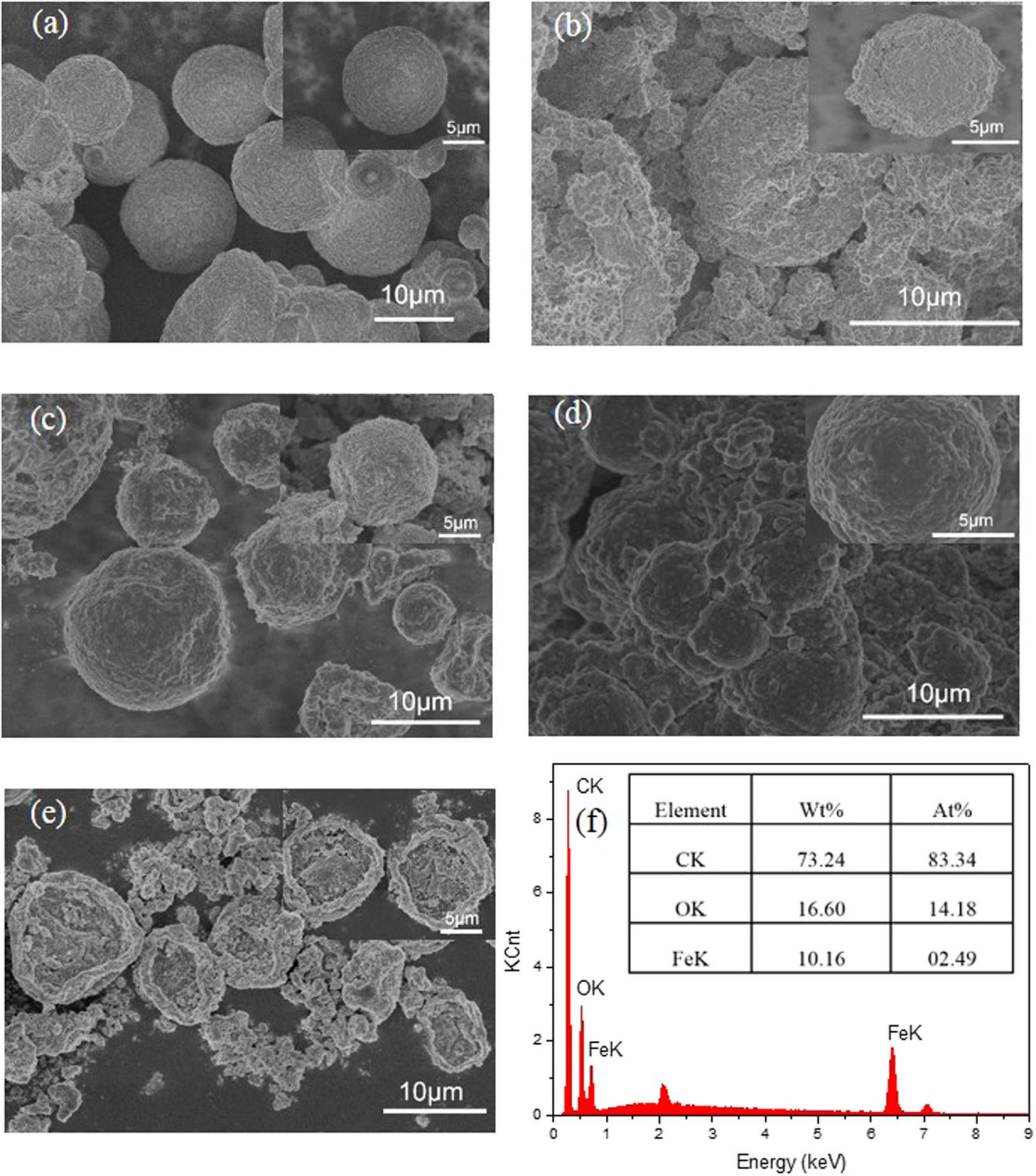
SEM micrographs of (**a**) MicroPCMs, magnetic MicroPCMs with Fe_3_O_4_ content of (**b**) 70%, (**c**) 20%, (**d**) 11%, (**e**) magnetic MicroPCMs destroyed with ultrasounds, and (**f**) EDX spectra of magnetic MicroPCMs.

There are four samples of different mass ratio of Fe_3_O_4_/MicroPCMs as controlling the amount of Fe^2+^ and Fe^3+^ in Table 2. And the SEM micrographs of the surface morphology of MicroPCMs and the magnetic MicroPCMs were presented in Fig. 4. Figure 4(a) shows the microscopic morphology of the MicroPCMs, they are excellent coated by Fe_3_O_4_ and the surface looks smooth and flat. The surface morphology of the magnetic MicroPCMs is different to MicroPCMs without magnetic particles, and it’s clearly shown in Fig. 4(b–d) that there are lots of nanoparticles on the MicroPCMs shell. Moreover, the magnetic MicroPCMs destroyed with ultrasounds still exhibits nanoparticles coated shell in Fig. 4(e). Therefore, the nanoparticles Fe_3_O_4_ could attach the surface of the MicroPCMs and the thin Fe_3_O_4_ presented better surface appearance. In addition, the EDX spectra of the magnetic MicroPCMs samples show peaks clearly corresponding to C, O and Fe elements in Fig. 4(f), confirming that the magnetic MicroPCMs shell consists of Fe_3_O_4_.

**Table 2:** Mass ratios of Fe_3_O_4_/MicroPCMs.

Sample No.	m(g, FeCl_2_·4H_2_O)	m(g, FeCl_3_·6H_2_O)	m(g, Fe_3_O_4_)	mass ratios of Fe_3_O_4_/MicroPCMs
1	2.99	1.35	4.67	70:30
2	0.88	0.6	0.5	20:80
3	0.44	0.3	0.25	11:89
4	0	0	0	0:100

**Figure 5:**
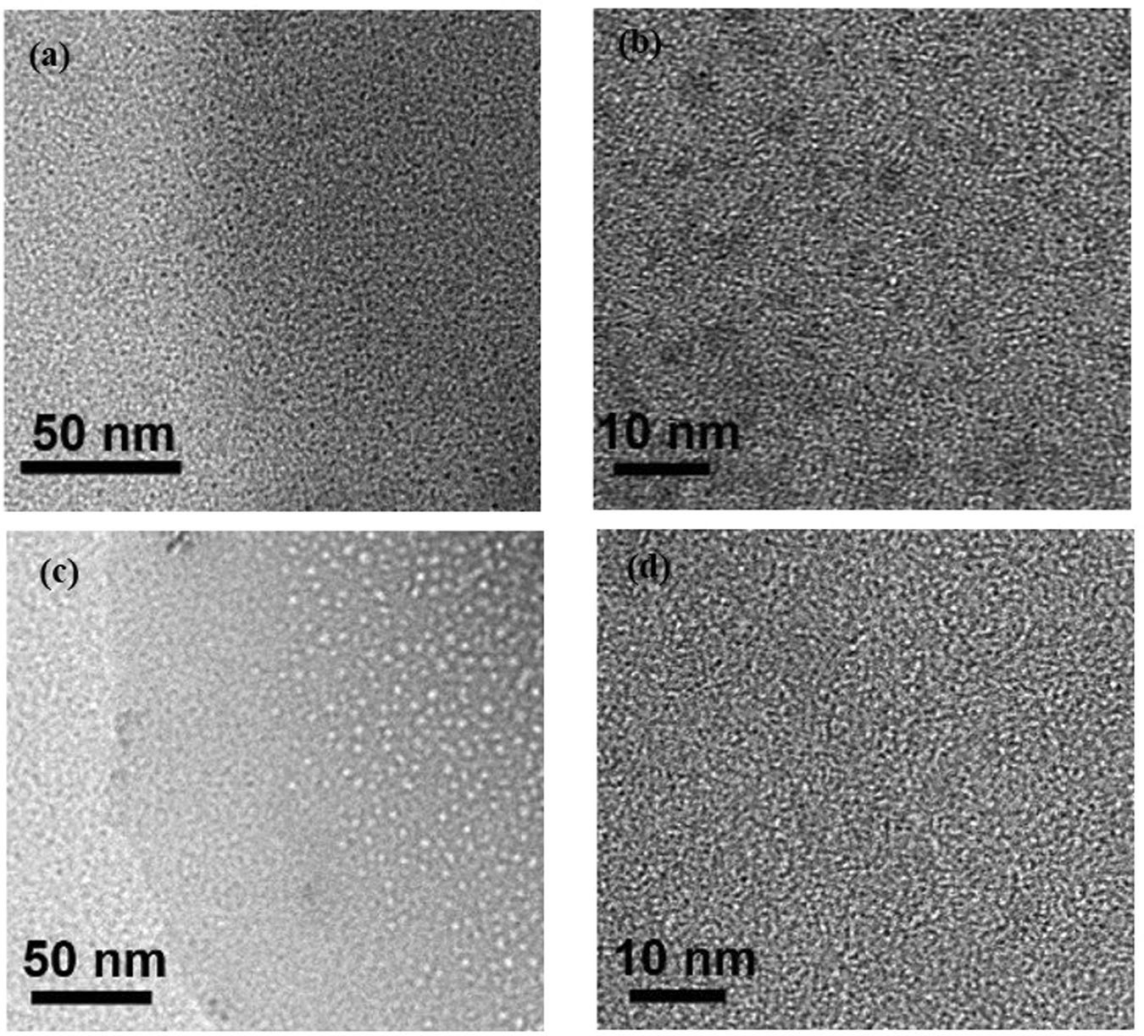
TEM micrographs of magnetic MicroPCMs of with Fe_3_O_4_/MicroPCMs mass ratios of (**a**) and (**b**) 20/80, and (**c**) and (**d**) 11/89.

The diameter and microstructures of Fe_3_O_4_ of the magnetic MicroPCMs obtained in this study were examined using HRTEM, and the resulting TEM micrographs were presented in Fig. 5. As is shown in Fig. 5(a,b), the border of the magnetic MicroPCMs with 20% Fe_3_O_4_ is not to distinguish clearly because the organic shell’s carbon element of the MicroPCMs is the same as micro grid of the sample preparation devices. Nevertheless, the Fe_3_O_4_ particles of the magnetic MicroPCMs can be discriminated distinctly with homogeneous distribution, and the size of Fe_3_O_4_ is at the nanoscale. The TEM micrographs of magnetic MicroPCMs with 11% Fe_3_O_4_ is also demonstrated as Fig. 5(c,d), the magnetic particles are more clear and uniform due to the less mass of Fe_3_O_4_. Meanwhile, the size and microstructure of Fe_3_O_4_ is same as 20% Fe_3_O_4_ of magnetic MicroPCMs.

**Figure 6:**
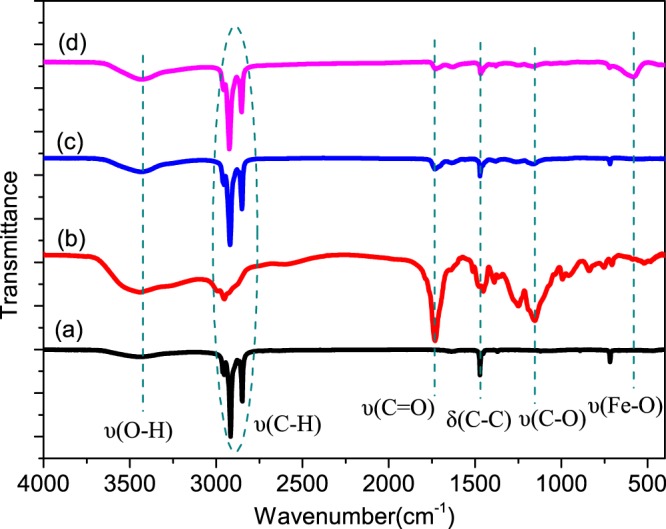
FTIR spectra of the (**a**) n-octadecane, (**b**) PMMA-MAA, (**c**) MicroPCMs, and (**d**) MicroPCMs-Fe_3_O_4._

The chemical structures and compositions of the magnetic MicroPCMs were characterized by FTIR spectroscopy and the resulting spectra are shown in Fig. 6, in which the infrared absorption spectra of the n-octadecane, ploy(MMA-MAA), and MicroPCMs are also presents. It is clearly known that 3430 cm^−1^ is ascribed to the O-H stretching vibrations peaks, three intensive absorption peaks at 2960 cm^−1^, 2920 cm^−1^ and 2850 cm^−1^ corresponding to the alkyl C-H stretching vibrations of methyl and methylene groups. Meanwhile, the absorption peak of 1732cm^−1^ is assigned to the C=O stretching vibrations of ester group, the peak at 1472 cm^−1^ denote C-H bending vibrations of methyl group, the peak at 1150 cm^−1^ is attributed to C-O stretching vibrations of ester group, and the peak Fe-O vibration appears at 575 cm^−1^ in the infrared spectra^34^. Compared to the three infrared absorption curves in Fig. 6(a–c), it is clearly noted that the vibration peaks of curve (a) and curve (b) can all be found in curve (c), which means that MicroPCMs is composed of the shell ploy(MMA-MAA) and the core n-octadecane. In addition, the curve (d) exhibits stronger Fe-O vibration peak than that in curve (c), it indicates that magnetic MicroPCMs has extra Fe_3_O_4_ than MicroPCMs. It proves that the magnetic MicroPCMs consists of n-octadecane, ploy(MMA-MAA) and Fe_3_O_4_.

**Figure 7:**
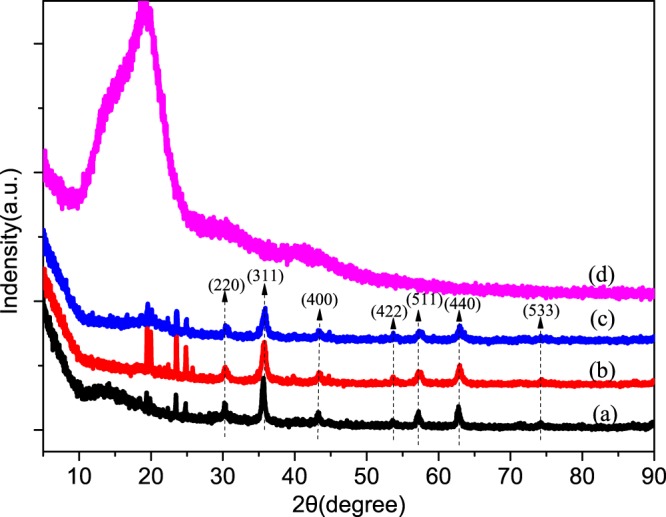
XRD patterns of the Fe_3_O_4_/MicroPCMs mass ratios of (**a**) 70/30, (**b**) 20/80, (**c**) 11/89, (**d**) 0/100.

The crystalline structure of the magnetic MicroPCMs shell was characterized by XRD, and the corresponding patterns were presented in Fig. 7. It is evidently shown the diffraction peaks at 2θ = 30.2°, 35.6°, 43.4°, 53.8°, 57.2°, 63.1°, and 74.2° in Fig. 7(a–c). According to the XRD standard PDF card no. 65-3107, these diffraction peaks are well attributed to the (220), (311), (400), (422), (511), (440), and (533) crystal planes of Fe_3_O_4_. However, the curve of Fig. 7(d) have not been found the diffraction peaks, it exactly explains the truth that the MicroPCMs shell poly(MMA-MAA) is amorphous. This well illustrated that the surface of magnetic MicroPCMs is coated by Fe_3_O_4_ nanoparticles.

It has been proved that the magnetic nanoparticles are coated on the shell of MicroPCMs from the SEM micrographs, EDX spectra, FTIR absorption spectra, and XRD pattern as mentioned above. This paper discussed the influences on the performance of MicroPCMs with different amounts of Fe_3_O_4_ below.

### The performance of MicroPCMs with different amounts of Fe_3_O_4_

The SEM micrographs of the surface morphology of the magnetic MicroPCMs with different dosages of Fe_3_O_4_ are illustrated in Fig. 4, and the corresponding sample number is shown in Table 2. It’s clearly to be found that the surface of the sample 1 is coated by lots of aggregative nanoparticles in Fig. 4(b), even they are easy to drop out from the surface, so that there are many nanoparticles fall out shown in Fig. 4(b). It is presented in Fig. 4(c) that the sample 2 have a complete coverage of the surface. Meanwhile, there are little nanoparticles out of the surface, it means that it is the proper dosage of the Fe_3_O_4_ on the sample 2. There are many magnetic MicroPCMs adhered to each other in the sample 3 was shown in Fig. 4(d). However, they are flatter on the surface than sample 2. Therefore, the little amounts of Fe_3_O_4_ could cover the surface of the magnetic MicroPCMs and make the surface smooth like the MicroPCMs. In addition, the distributions of the particles diameter with different mass of Fe_3_O_4_ were given in Fig. 3(d–f). And the average diameter of the magnetic MicroPCMs with 70% Fe_3_O_4_ has a great increase from 11 to 13 μm. However, the average diameter of the sample 2 and 3 just have a little increase. These prove that the small amount of Fe_3_O_4_ could attach with shell of MicroPCMs densely. Furthermore, the diameters of the magnetic MicroPCMs confirm that Fe_3_O_4_ particles covered the surface of MicroPCMs uniformly. To conclude, 20 percent of Fe_3_O_4_ in the magnetic MicroPCMs can present excellent microscopic surface morphology.

#### Phase change characteristics and thermal stability performance

**Figure 8:**
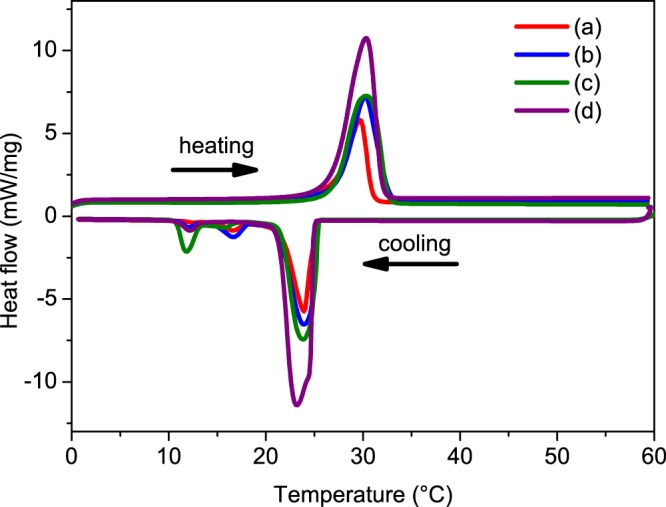
DSC cooling and heating thermograms of the Fe_3_O_4_/MicroPCMs mass ratios of (**a**) 70/30, (**b**) 20/80, (**c**) 11/89, (**d**) 0/100.

The phase change behaviors were investigated by DCS and the resulting DSC thermograms are presented in Fig. 8. Table 3 lists the thermal properties of MicroPCMs with various Fe_3_O_4_ dosage, in which Δ*H*_*ac*_ means the calculate value of the enthalpy with different amounts of Fe_3_O_4_ by ${\rm{\Delta }}{H}_{ac}={\rm{\Delta }}{H}_{aMicroPCMs}\times {w}_{MicroPCMs}$ and the *w*_*MicroPCMs*_ means the content of MicroPCMs in magnetic MicroPCMs. Compared to the four curves in Fig. 8, there are not great influence on melting peaks and the crystalline peaks, and they all have the melting peaks at about 30 °C and the crystalline peaks at about 24 °C. Meanwhile, the average enthalpy Δ*H*_*a*_ decrease with increase of the Fe_3_O_4_ amounts. It is clearly known that the Δ*H*_*a*_ is related to the content of the n-octadecane, and the increased amounts of Fe_3_O_4_ must lead to the decrease of the n-octadecane’s content in the magnetic MicroPCMs. In addition, the Δ*H*_*a*_ are pretty close to the Δ*H*_*ac*_ of the sample 2 and sample 3, it means that the results is corresponding to the anticipation. However, the sample 1 presents the great difference between Δ*H*_*a*_ and Δ*H*_*ac*_, the value Δ*H*_*a*_ is greater than Δ*H*_*ac*_ in Table 3. It indicates that there are some Fe_3_O_4_ nanoparticles lost in the fabrication process of the magnetic MicroPCMs, so that the actual quality of Fe_3_O_4_ is lower than the expected value. Nonetheless, the 20 percent content of Fe_3_O_4_ nanoparticles in the magnetic MicroPCMs could coated the MicroPCMs well and still keep high enthalpy.

**Table 3:** Thermal properties of MicroPCMs with various Fe_3_O_4_ dosage.

Sample No.	Fe_3_O_4_: MicroPCMs	T_m_ (°C)	T_c_ (°C)	Δ*H*_*a*_ (J·g^−1^)	Δ*H*_*ac*_ (J·g^−1^)	n-octadecane (wt.%)
1	70:30	30.3	23.5	88	51.3	37.0
2	20:80	30.3	23.5	132	136.8	55.5
3	11:89	30.3	23.5	154	152.2	64.7
4	0:100	30.3	23.5	171	171	71.9

**Figure 9:**
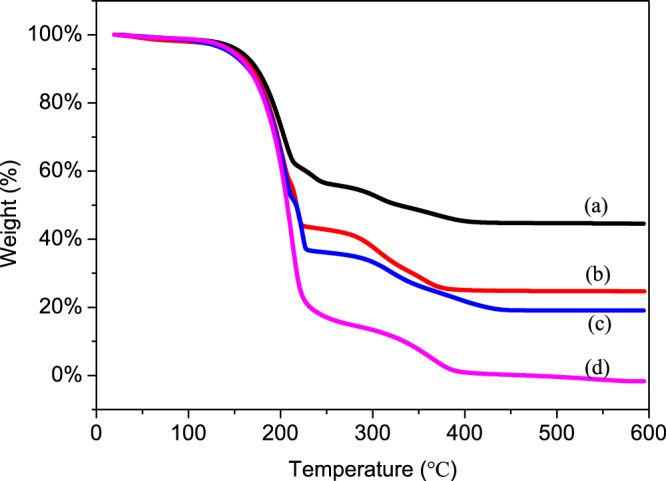
TGA thermograms of the Fe_3_O_4_/MicroPCMs mass ratios of (**a**) 70/30, (**b**) 20/80, (**c**) 11/89, (**d**) 0/100.

The thermal stability of the magnetic MicroPCMs was investigated by TGA and the resulting thermograms are presented in Fig. 9. The MicroPCMs starts to loss the weight at about 180 °C in Fig. 9(d), it is clearly shown that high temperature make the MicroPCMs swell, n-octadecane flows out of the MicroPCMs and begins to decompose. The second weight loss happens at nearly 300 °C, because the polymer shell starts to decompose with the molecular chain ruptured. The weight loss curves of magnetic MicroPCMs are presented in Fig. 9(a–c). There is the second weight loss at around 200 °C differs from MicroPCMs, it is obviously known that the Fe_3_O_4_ nanoparticles coated on the surface lead to high temperature of the weight loss for some n-octadecane. The first and third weight loss of the magnetic MicroPCMs are the same temperature as the first and second weight loss of MicroPCMs. Hence, compare with MicroPCMs, the magnetic MicroPCMs have a certain degree of ascension. In addition, because the pure n-octadecane decomposition temperature (T_d_) is just 128 °C^40^, the thermal stability of magnetic MicroPCMs has a substantial increase.

#### Magnetic properties

**Figure 10:**
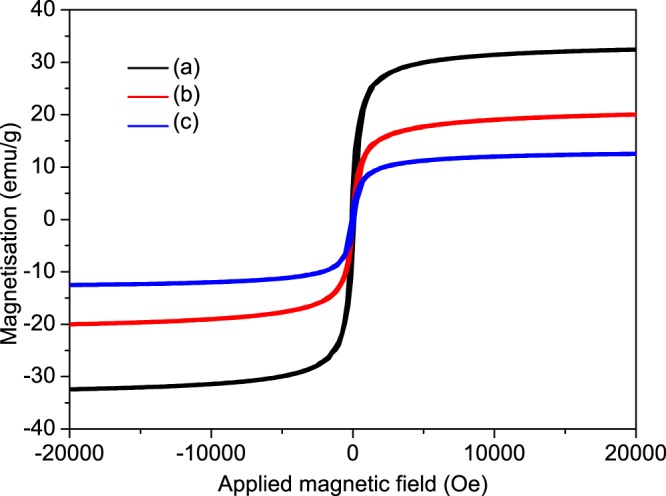
Magnetic hysteresis loops of the Fe_3_O_4_/MicroPCMs mass ratios of (**a**) 70/30, (**b**) 20/80, (**c**) 11/89.

The magnetic properties of the magnetic MicroPCMs were characterized by PPMS-9 at room temperature (~300 K), and the resulting magnetic hysteresis loops are shown in Fig. 10. It is obviously shown that three samples exhibit low residual intensity of magnetization and coercivity. It means that the magnetic MicroPCMs possess super paramagnetic behavior. Furthermore, the saturation magnetization of three magnetic MicroPCMs samples were measured to be 32.5 emu·g^−1^, 20 emu·g^−1^, 12.5 emu·g^−1^ when the Fe_3_O_4_/MicroPCMs mass ratios of 70/30, 20/80, and 11/89, correspondingly. It is clear that the saturation magnetization improved with the increase of Fe_3_O_4_ content. This further proves that the Fe_3_O_4_ could well coated on the surface of the MicroPCMs with various amounts.

## Conclusions

In this work, we coated the Fe_3_O_4_ nanoparticles on the surface of the MicroPCMs and fabricated the functional phase change materials with magnetic property. A series of products were prepared with differ amounts of Fe_3_O_4_ and characterized by particle size analyzer for size distributions, SEM and TEM for microscopic morphology, FTIR for chemical structure, XRD for crystalline structure, DSC for phase change behaviors, TGA for thermal stability, and PPMS for magnetic properties. After these characterizations, the magnetic MicroPCMs exhibits excellent magnetic and phase change properties. For example, the 11 percent of content of Fe_3_O_4_ in the magnetic MicroPCMs still present 20 emu·g^−1^ saturation magnetization with 132 J·g^−1^ enthalpy. This will provide new thinking on the applications of the phase change materials on electromagnetic protection clothing with thermostat for soldiers, gravida, or other special operations personnel, infrared-electromagnetic dual shield for stealth aircraft, and thermal manipulation in real production.
